# UCP1 Knockin Induces Lipid Dynamics and Transcriptional Programs in the Skeletal Muscles of Pigs

**DOI:** 10.3389/fcell.2021.808095

**Published:** 2022-01-12

**Authors:** Ziye Xu, Wentao Chen, Liyi Wang, Wenjing You, Yanfang Wang, Yizhen Wang, Jianguo Zhao, Tizhong Shan

**Affiliations:** ^1^ College of Animal Sciences, Zhejiang University, Hangzhou, China; ^2^ Key Laboratory of Molecular Animal Nutrition, Ministry of Education, Zhejiang University, Hangzhou, China; ^3^ Key Laboratory of Animal Feed and Nutrition of Zhejiang Province, Zhejiang University, Hangzhou, China; ^4^ State Key Laboratory of Animal Nutrition, Institute of Animal Science, Chinese Academy of Agricultural Sciences, Beijing, China; ^5^ Institute of Zoology, Chinese Academy of Sciences, Beijing, China

**Keywords:** UCP1-KI, lipidomics, transcriptome, skeletal muscle, pig, crosstalk, adipose tissue, obesity

## Abstract

Uncoupling protein 1 (UCP1), the hallmark protein responsible for nonshivering thermogenesis in adipose tissue (especially brown adipose tissue) has regained researchers’ attention in the context of metabolic disorders following the realization that UCP1 can be activated in adult humans and reconstituted in pigs. Both skeletal muscle and adipose tissue are highly dynamic tissues that interact at the metabolic and hormonal level in response to internal and external stress, and they coordinate in maintaining whole-body metabolic homeostasis. Here, we utilized lipidomics and transcriptomics to identify the altered lipid profiles and regulatory pathways in skeletal muscles from adipocyte-specific UCP1 knock-in (KI) pigs. UCP1 KI changed the contents of glycerophospholipids and acyl carnitines of skeletal muscles. Several metabolic regulatory pathways were more enriched in the UCP1 KI skeletal muscle. Comparison of the transcriptomes of adipose and skeletal muscle suggested that nervous system or chemokine signaling might account for the crosstalk between these two tissues in UCP1 KI pigs. Comparison of the lipid biomarkers from UCP1 KI pigs and other mammals suggested associations between UCP1 KI-induced metabolic alternations and metabolic and muscle dysfunction. Our study reveals the lipid dynamics and transcriptional programs in the skeletal muscle of UCP1 KI pigs and suggests that a network regulates metabolic homeostasis between skeletal muscle and adipose tissue.

## Introduction

Uncoupling protein-1 (UCP1) is the hallmark protein of non-shivering thermogenesis and allows brown adipose tissue (BAT) in mammals to alter energy expenditure and lipid metabolism in response to environmental stress (Fromme and Klingenspor, 2011). Furthermore, UCP1-positive browning white adipocytes or beige cells have the ability to adopt a BAT-like thermogenic phenotype in response to various stimuli including cold stress, endocrine factors, and chemical compounds (Barbatelli et al., 2010; Cao et al., 2011; Barquissau et al., 2016; Kiefer, 2016). The potentially underappreciated role of UCP1 in human metabolic health have attracted much attention recently, largely in response to *in vivo* demonstrations and activation of functional BAT in human adults in preclinical studies. Several studies have revealed that BAT activation increases glucose uptake and improves whole-body glucose disposal and insulin sensitivity in healthy humans or overweight men with type 2 diabetes (Orava et al., 2011; Ouellet et al., 2012; van der Lans et al., 2013; Yoneshiro et al., 2013; Matsushita et al., 2014; Hanssen et al., 2015). What’s more, emerging evidence has shown that manipulation of BAT function has implication for skeletal muscle physiology and thermogenesis by using surgical ablation of BAT (Bal et al., 2016) and UCP1-KO mice (Bal et al., 2017). However, although the roles of UCP1 in whole body metabolism and the associated regulatory mechanisms have been extensively studied in rodents (Bartelt et al., 2011; Laurila et al., 2016), the role of UCP1 in humans have yet to be fully elucidated and require further study. Pigs have been recognized as valuable animal models for studying obesity and diabetes mellitus (Koopmans and Schuurman, 2015; Renner et al., 2016; Kleinert et al., 2018). However, pigs lack abundant BAT or UCP1 protein during evolution (Berg et al., 2006; Hou et al., 2017; Lin et al., 2017). In our previous study, we generated adipocyte-specific UCP1 knock-in (UCP1-KI) pigs by CRISPR/Cas9 technology and revealed a critical role of UCP1 in maintaining metabolic homeostasis in pigs (Zheng et al., 2017).

Skeletal muscle has been considered as a metabolically flexible and promiscuous organ because of its complex components (muscle fibers, intramuscular connective tissue, and intramuscular fat) and strong propensity for glucose disposal and fatty acid consumption during the development of several metabolic disorders, such as obesity and type 2 diabetes mellitus (T2DM) (Alberti and Zimmet, 1998; McFarlan et al., 2012; Morales et al., 2017). Different lines of evidence have indicated that insulin resistance is associated with lipid species alterations inside skeletal muscle, such as intramyocellular lipids (Krssak et al., 1999), ceramide (Cer) (Holland et al., 2007) and diacylglycerol (Itani et al., 2002; Yu et al., 2002). Thus, novel therapeutic strategy for preventing and/or treating metabolism-related diseases by targeting lipids (such as Cers) in skeletal muscle have been proposed and investigated (Schmitz-Peiffer et al., 1999; Powell et al., 2004; Aburasayn et al., 2016). The lipid composition of skeletal muscle from domesticated animals, including pigs, cattle and chickens, is one of the most critical influences on meat quality and nutritional value, which are increasingly important factors influencing consumer preferences for meat and meat products (Scollan et al., 2017). Thus, interest in the development of methods to regulate the lipid content and composition of skeletal muscle has been increasing. It has been reported that the metabolic characteristics and lipid composition of skeletal muscle can be regulated by several adipose-released hormones and adipokines (Tomas et al., 2004; Zhou et al., 2007). Furthermore, it has been shown that whole-body metabolic homeostasis is usually coordinately regulated by skeletal muscle and adipose tissue through the interactions of cytokines and other signaling molecules (Tomas et al., 2004; Argiles et al., 2005; Li et al., 2017; Rodriguez et al., 2017).

The metabolic characteristics and lipid deposition of skeletal muscle are affected by several factors, including hormones, nutritional factors, exercise and environmental factors. Notably, crosstalk between skeletal muscle and adipose tissue has been identified that regulates whole-body metabolic homeostasis via myokines and adipokines (Argiles et al., 2005; Li et al., 2017; Rodriguez et al., 2017; Tomas et al., 2004). In addition to supplying skeletal muscle with free fatty acids (FFAs), adipose tissue releases several hormones and adipokines to modulate the metabolism and function of skeletal muscle (Tomas et al., 2004). It has been demonstrated that the adipokines adiponectin and leptin can activate AMP-activated protein kinase and increase FA oxidation in skeletal muscle (Tomas et al., 2004). Furthermore, adiponectin and FFAs have impacts on muscle proteolysis under insulin-resistance conditions (Zhou et al., 2007). Adipose tissues, especially brown adipose tissue, act in concert through a number of cellular pathways to regulate energy metabolism and nonshivering thermogenesis (Fuller-Jackson and Henry, 2018). However, little is known about the effect of adipose tissue on the metabolism of skeletal muscle under nonshivering thermogenesis conditions.

Our previous study indicated that UCP1 KI pigs exhibited improved thermoregulation upon cold exposure and dramatically decreased fat deposition due to elevated lipolysis in inguinal white adipose tissue (iWAT) (Zheng et al., 2017). In addition, it revealed enhanced mitochondrial function in the adipose tissue from UCP1 KI pigs by lipidomics and RNA-seq analysis (Pan et al., 2019), and a specific role of UCP1 in the lipid metabolism of fat tissues in pigs. However, whether adipocyte-specific UCP1 KI elicits metabolic changes in other organs, such as skeletal muscle, remains unclear. Considering the close association between skeletal muscle and adipose tissue, we hypothesized that adipocyte-specific KI UCP1 may influence lipid metabolism and skeletal muscle function. In the present study, we conducted lipidomics and transcriptomics on skeletal muscle from 6-month-old UCP1 KI and wild-type (WT) pigs to explore the lipid dynamics and transcriptional programs induced by adipocyte-specific UCP1 overexpression in skeletal muscle. In addition, we mapped the transcriptional profiles and lipid-metabolic regulatory pathways in skeletal muscle from UCP1 KI pigs. Moreover, we compared the genetic and lipidic profiles between adipose and skeletal muscle tissue from UCP1 KI pigs, and we compared the significantly altered lipids identified in UCP1 K1 pigs with those identified in humans and mice with metabolic or muscle dysfunction. Our work establishes a network of the regulatory pathways involved in metabolic homeostasis and dynamics in skeletal muscle through adipocyte-specific KI of UCP1 in pigs and reveals a novel interaction between skeletal muscle and adipose tissue.

## Materials and Methods

### Animals and Samples

All experiments involving animals were conducted according to the guidelines for the care and use of laboratory animals established by the Beijing Association for Laboratory Animal Science and approved by the Animal Ethics Committee of the Institute of Zoology, Chinese Academy of Sciences. The animals used in the experiment included WT pigs and adipocyte-specific UCP1 KI pigs. Adipocyte-specific UCP1 KI pigs were generated as described previously (Zheng et al., 2017). The samples of skeletal muscle tissues were dissected from the longissimus dorsi muscle frozen immediately in liquid nitrogen, and stored at −80°C until use.

### Lipid Sample Preparation and Lipidomics Assay

Lipid extraction and mass spectrometry-based lipid detection of skeletal muscle samples from WT and adipocyte-specific UCP1 KI pigs were performed by Applied Protein Technology using previously published methods (Xu et al., 2019). Briefly, samples from each group were mixed together to create a pooled QC sample. QC samples were inserted into the analysis queue to evaluate the system stability and data reliability during the entire experimental process. LC-MS/MS analysis was performed on a Q Exactive plus mass spectrometer (Thermo Scientific) coupled to a UHPLC Nexera LC-30A instrument (SHIMADZU). Full-scan spectra were collected in mass-to-charge ratio (m/z) ranges of 200–1,800 and 250–1,800 for positive- and negative-ion modes, respectively. The mass-to-charge ratio of lipid molecules to lipid fragments was determined by the following method: after each full scan, 10 fragment patterns (MS2 scan, high-energy collision dissociation (HCD)) were collected. Lipid identification (secondary identification), peak extraction, peak alignment, and quantification were assessed by LipidSearch software version 4.1 (Thermo Scientific™). Regarding the extracted ion features, only those variables having more than 50% nonzero values in at least one group were kept retained. Identified lipids were subjected to unsupervised multivariate analyses and analysis of fatty acyl chain compositions.

### Unsupervised Multivariate Data Analyses

SIMCA-P 14.1 software (Umeta, Umea, Sweden) was utilized for the multivariate statistical analysis. After Pareto scaling, PCA, partial least-squares-discriminant analysis (PLS-DA) and OPLS-DA were performed. Leave-one-out cross-validation and response permutation testing were used to evaluate the robustness of the model. The significantly different metabolites were determined based on the significant threshold VIP value from the PLS-DA model and two-tailed Student’s *t*-test (*p*-value) with a VIP value larger than 1.0 and a *p*-value less than 0.05 identifying a metabolite as significantly different. Univariate analyses included Student’s *t*-test and variable fold-change analysis. Hierarchical cluster analysis and correlation analysis were performed with the function heatmap () and cor (), respectively, in R software (version 3.5.1).

### RNA Isolation, Library Construction and RNA-seq Analysis

RNA extraction, library construction and RNA-seq analysis of skeletal muscle samples from WT and adipocyte-specific UCP1 KI pigs were performed by Sangon Biotech using previously published methods (Xu et al., 2019). Total RNA was extracted using the Total RNA Extractor (TRIzol) Kit (B511311, Sangon, China) according to the manufacturer’s protocol and treated with RNase-free DNase I to remove genomic DNA contamination. A total amount of 2 μg RNA per sample was used as input material for subsequent RNA sample preparations. Sequencing libraries were generated using the VAHTSTM mRNA-seq V2 Library Prep Kit for Illumina® following the manufacturer’s recommendations, and index codes were added to attribute sequences to each sample. The libraries were then quantified and pooled. Paired-end sequencing of the library was performed on HiSeq XTen sequencers (Illumina, San Diego, CA). The quality of the sequenced data was evaluated by FastQC (version 0.11.2). Trimmomatic (version 0.36) was applied to filter raw reads and clean reads, were mapped to the reference genome by HISAT2 (version 2.0) with the default parameters. RSeQC (version 2.6.1) was used to analyze the alignment results. The homogeneity distribution and genomic structure were evaluated by Qualimap (version 2.2.1). BEDTools (version 2.26.0) was applied for statistical analysis of the gene coverage ratio. StringTie (version 1.3.3b) was used to compute the gene expression values of the transcripts. The TPM, which eliminated the influences of gene length and sequencing discrepancies, was applied for direct comparison of gene expression between samples. The DESeq2 package (version 1.12.4) of R software (version 3.5.1) was applied to the gene count data to determine DEGs between the two groups. Genes with values <0.05 and |Log2(fold change) |>0.5 were considered significant DEGs.

### Pathway-Enrichment Assay

The identified DEGs were subjected to GO functional analysis and KEGG pathway analysis using the packages clusterProfiler and org.Ss.eg.db in R software (version 3.5.1). DEGs were mapped to the GO terms (in the biological functions categories) in the database. The number of genes in every term was calculated, and a hypergeometric test was performed to identify significantly enriched GO terms in the gene list out of the background of the reference-gene list. Enriched terms were visualized by barplot and a network of the most enriched terms was constructed and visualized by Cnetplot. KEGG pathway analysis identified significantly enriched metabolic pathways or signal transduction pathways enriched in DEGs compared to those of a reference gene background using the hypergeometric test. Enriched pathways were visualized by dotplot.

### Correlational Assay

Correlations were calculated by function cor() in R software (version 3.5.1) using the method “spearman” or “pearson” for the Spearman’s or Pearson’s correlation coefficient analysis, respectively. Pearson’s correlation is a measure of linear correlation, while Spearman’s correlation is based on the ranks of values rather than the values themselves.

### Data Analysis

All the statistical evaluations of lipidomics data described in this work were calculated from relative abundances. The data are presented as the mean ± SEM. Comparisons were performed by unpaired two-tailed Student’s *t*-test or one-way ANOVA, as appropriate. Differences among groups were considered statistically significant at *p* < 0.05.

## Results

### Adipocyte-Specific UCP1 KI Alters the Overall Composition of Lipid Classes in Skeletal Muscle

**FIGURE 1:**
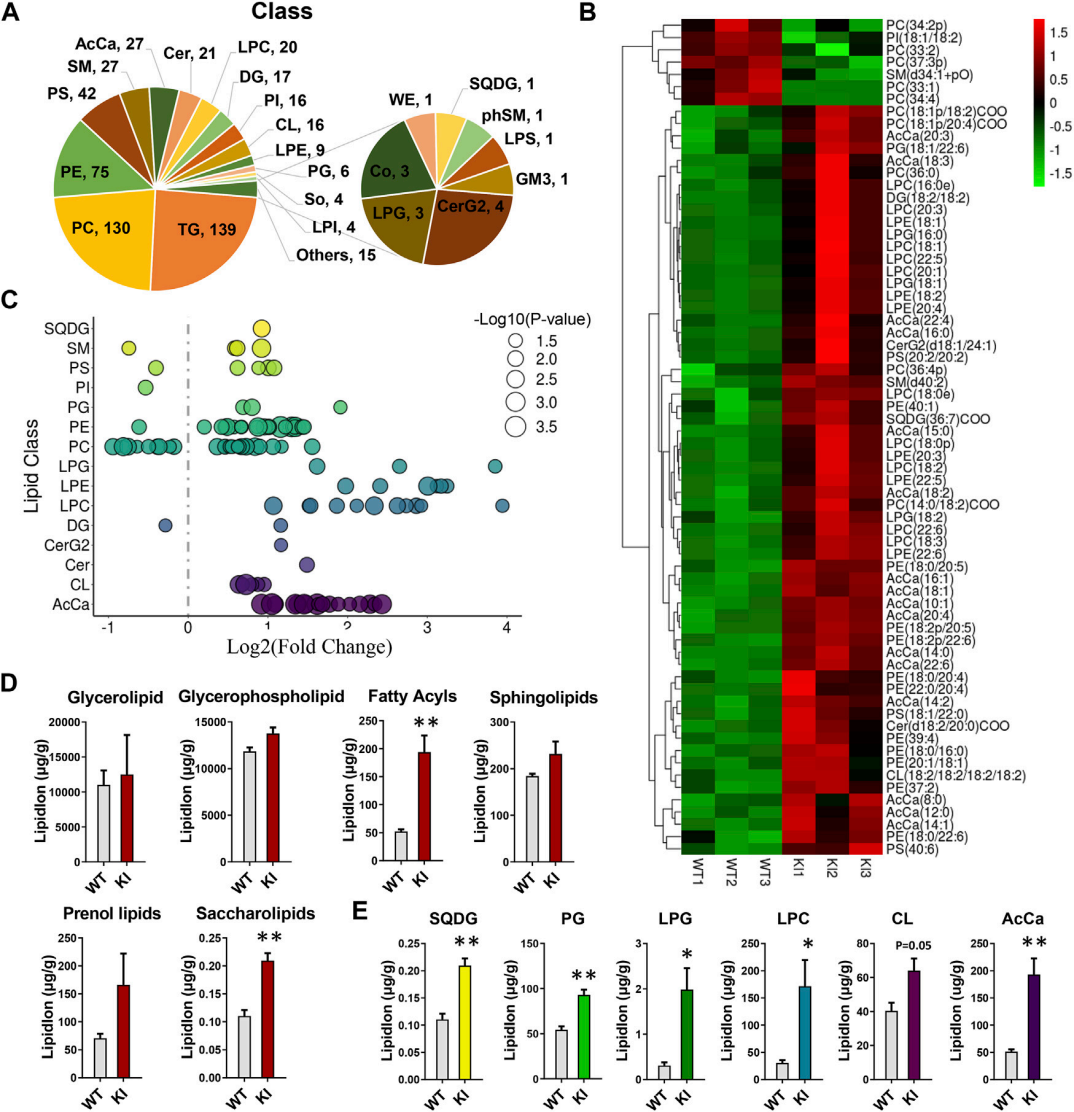
Changes in the overall lipid composition and distribution in skeletal muscle induced by adipocyte-specific expression of UCP1. Lipidomics analysis of skeletal muscle from UCP1 KI pigs. Lipids were extracted and analyzed as described in the *Materials and Methods*. **(A)** Distribution of lipid classes that were considered for subsequent analysis in all of the samples detected by LC-MS/MS. **(B)** Heatmap of the significantly altered lipids (*p*-value <0.05 and VIP >1) in skeletal muscle from controls and UCP1 KI pigs (*n* = 3). **(C)** Log2 fold changes in lipid species in the KI- versus WT- group, and the corresponding significance values are displayed as −log10 (*p*-value). Each dot represents a lipid species, and the dot size indicates significance. Only lipids with *p* < 0.05 are displayed (*n* = 3). **(D)** The lipid ion intensity of lipid groups, including glycerolipids, GLs, fatty acyls, sphingolipids, prenol lipids and saccharolipids. **(E)** The lipid ion intensity of significantly changed lipid classes, including SQDG, PG, LPG, LPC, CL and AcCa. Data are presented as the means ± SEM (*n* = 3). *p < 0.05, ***p < 0.001.

To determine the effects of adipocyte-specific UCP1 overexpression on the overall lipid composition and distribution in skeletal muscle, we isolated skeletal muscle from adipocyte-specific UCP1 KI pigs and performed liquid chromatography tandem mass spectrometry (LC-MS/MS) analysis. We detected over 568 different lipid species in skeletal muscle, comprising 139 triglycerides (TGs), 130 phosphatidylcholines (PCs), 75 phosphatidylethanolamines (PEs) and other lipid classes (Figure 1A). The abbreviations of these quantified lipid classes are shown in Supplementary Figure S1A. Multivariate analysis (MVA) using PCA showed a clear separation of the UCP1 KI and WT groups (Supplementary Figure S1B). Both projections to latent structures discriminant analysis (PLS-DA) and orthogonal projections to latent structures discriminant analysis (OPLS-DA) plots distinguished two clusters (Supplementary Figures S1C,D). Seventy-two important variables ((acyl carnitine (AcCa) (10:1), PE (18:2p/22:6), AcCa (16:1) and others) were selected for separating UCP1 KI and WT groups based a variable influence in projection (VIP) value >1.0 and *p*-value <0.001 in the OPLS model. These variables might be considered biomarkers for skeletal muscle from UCP1 KI pigs. The changes in these 72 features in WT and UCP1 KI samples were visualized by heatmap (Figure 1B), and their correlations are shown in Supplementary Figure S2. The majority of these 72 features were glycerophospholipids (GLs) or AcCas and were obviously elevated by adipocyte-specific UCP1 overexpression, with high positive correlations with each other. To explore individual lipid species that differed between the WT and KI groups, all of the significantly changed lipid species were visualized using a bubble map. Using a *p*-value of 0.05 as a cutoff, a total of 109 species in skeletal muscle were identified as significantly changed in UCP1 KI pigs (Figure 1C). Analyses of lipid groups showed that the total contents of fatty acyls and saccharolipids were significantly increased in the UCP1 KI group relative to the WT group (Figure 1D), whereas no significant differences in the total contents of glycerolipids, GLs, sphingolipids and prenol lipids were found between groups (Figure 1D). In addition, we found that UCP1 KI significantly increased the contents of the following lipid classes: sulfoquinovosyldiacylglycerols (SQDGs), phosphatidylglycerols (PGs), lysophosphatidylglycerols (LPGs), lysophosphatidylcholines (LPCs), AcCas and cardiolipins (CLs) (*p* = 0.05) (Figure 1E). These findings suggest that UCP1 KI in pigs induces considerable alterations in the composition and content of lipid species in skeletal muscle.

### Adipocyte-Specific Uncoupling Protein 1 Knock-in Regulates the Composition of Fatty Acyl Chains Associated With Glycerophospholipids

TGs and GLs were the two most abundant lipids in skeletal muscle. The fatty-acyl chains associated with TGs and GLs reflect the composition of major fatty acids in skeletal muscle. The results showed that the contents of total TGs and TG lipid species showed no significant changes between KI and WT pigs. Consistently, none of the fatty acyl chains associated with TGs was significantly changed in skeletal muscle from UCP1 KI pigs (Supplementary Figures S3A–D), and neither were the total percentages of saturated fatty acyl (SFA) chains, monounsaturated fatty acyl (MUFA) chains, and polyunsaturated fatty acyl (PUFA) chains associated with TG acyl chains (Supplementary Figure S3E). Most of the carbon numbers and double bonds of the TG acyl chains showed an increasing trend (Supplementary Figures S3F,G).

**FIGURE 2:**
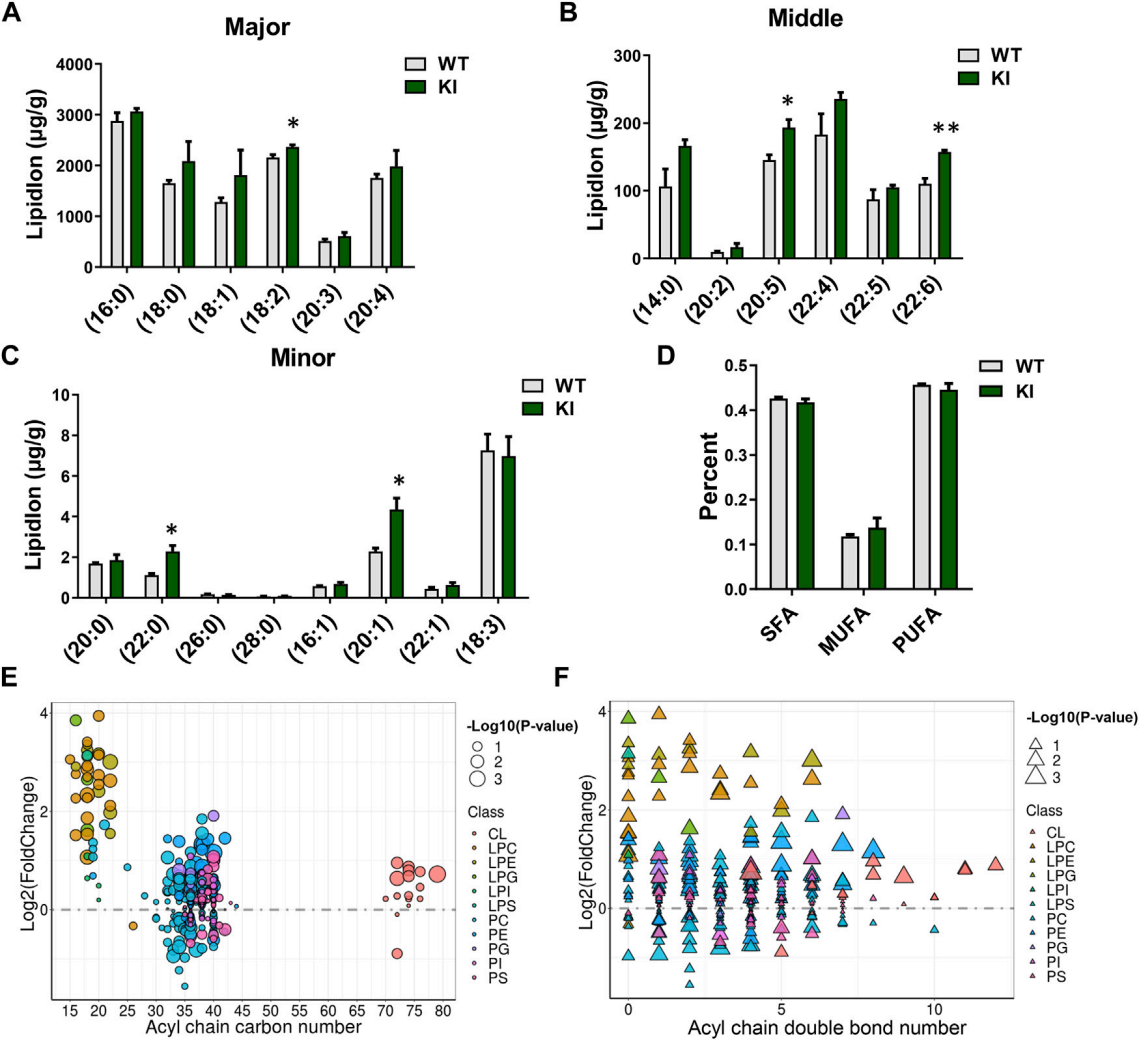
Adipocyte-specific UCP1 KI changed the composition of fatty acyl chains associated with GLs in skeletal muscle. **(A–C)** The total lipid ion intensity of individual fatty acyl chains associated with GLs sorted by degree of intensity. Data are presented as means ± SEM (*n* = 3). *p < 0.05, ***p < 0.001. **(D)** The total percentages of SFA chains, MUFA chains and PUFA chains associated with GLs. SAF, saturated fatty acyls; MUFA, monounsaturated fatty acyls; PUFA, polyunsaturated fatty acyls containing two or three to six double bonds. **(E,F)** The GL pattern in cold-treated cases versus that in controls. Each dot or triangle represents a distinct GL, organized along the x-axis based on the total acyl chain carbon number **(E)** or double bond content **(F)**. The size of each dot or triangle is proportional to the significance values, which are displayed as −log10 (*p*-value). The different colors of each dot or triangle represent each lipid classes, including CL, LPC, LPE, LPG, LPI, LPS, PC, PE, PG, PI, and PS.

We next analyzed the individual fatty-acyl-chain composition associated with GLs. Significant increases in the concentrations of SFA chains (C22:0), MUFA chains (C20:1) and PUFA chains (C18:2, C20:5, C22:6) in the GL pool were found in the UCP1 KI group relative to the WT group (Figures 2A–C). Notably, the content of DHA (C22:6) associated with GLs in the UCP1 K1group was dramatically increased compared to that in the WT group; DHA is widely believed to be highly beneficial to human physiology (Figure 2B). However, the percentages of SFA chains, MUFA chains and PUFA chains associated with GLs were not significantly different between the WT and KI groups (Figure 2D). Relative to that from WT pigs, skeletal muscle from UCP1 KI pigs displayed markedly elevated levels of GL species with lower (<25 carbons, mostly associated with LPCs, LPEs, LPGs, LPIs and LPSs) or higher (>70 carbons, associated with CLs) acyl-chain carbon numbers (Figure 2E). Half of the GL species with middle acyl-chain carbon numbers (>25 carbons and <70 carbons, mostly associated with PC, PE, PG, PI, PS) were increased in the KI group, while the others were decreased in this group (Figure 2E). Most of the GL species with fewer (<2, mostly associated with LPCs and LPEs) acyl-chain double bonds had significantly elevated levels in the KI group relative to the WT group (Figure 2F). The fatty acid composition reflected by GLs indicated significant alterations in the skeletal muscle of UCP1 KI pigs relative to that from WT pigs.

### Adipocyte-Specific Uncoupling Protein 1 Knock-in Changes the Composition of Fatty Acyl Chains Associated With AcCas

**FIGURE 3:**
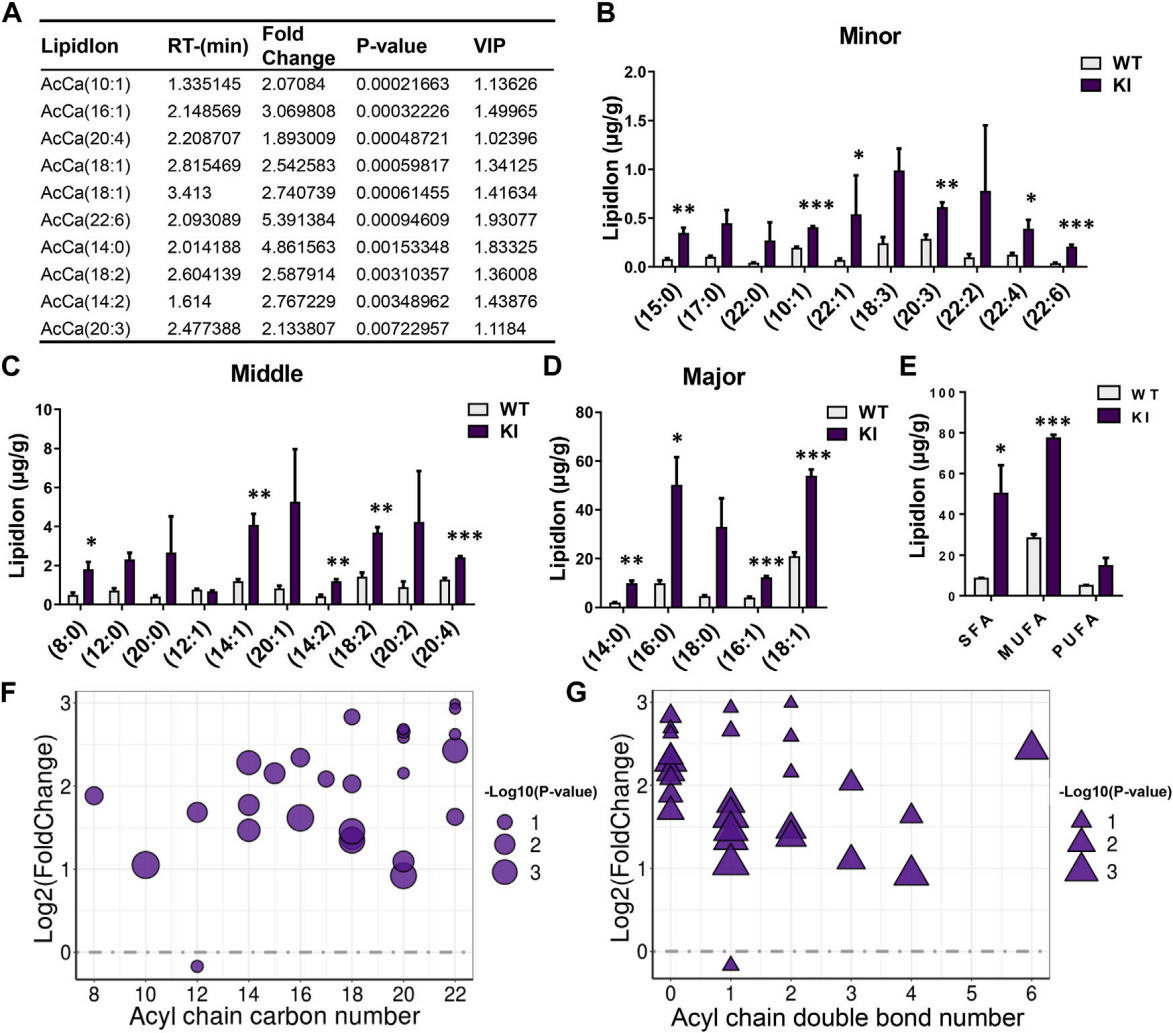
UCP1 KI changed the composition of fatty acyl chains associated with AcCas in skeletal muscle. **(A)** The top 10 AcCas according to the P-value, detected in skeletal muscle from UCP1 KI pigs and controls (*n* = 3). **(B–D)** The total lipidIon intensity of individual Fatty acyl chains associated with AcCas sorted by degree of intensity. **(E)** The total lipid ion intensity of SFA, MUFA and PUFA associated with AcCas. Data are presented as means ± SEM (*n* = 3). *p < 0.05, ***p < 0.001. **(F,G)** The AcCas pattern in cold-treated cases versus that in controls. Each dot or triangle represents a distinct AcCas, organized along the x axis based on total acyl chain carbon number **(F)** or double bond content **(G)**. The size of each dot or triangle is proportional to the significance values, which are displayed as −log10 (*p*-value).

As the above described results showed that adipocyte-specific UCP1 KI significantly changed the total content of AcCas, which are important diagnostic markers for several peroxisome and mitochondria-related metabolic syndrome (Ramsay et al., 2001), we analyzed the composition of individual AcCa species and fatty acyl chains associated with AcCas. We ranked AcCa lipids according to *p*-values, compared them between the KI and WT groups, and examined the top 10 species (Figure 3A). A greater than 1.5 fold change was found between the KI and WT groups in the top 10 AcCas, of which one was a SFA, four were MUFAs and five were PUFAs (Figure 3A). In line with the alteration of C22:6 associated with GLs, AcCa (C22:6) exhibited the highest fold change (5.39), being elevated in KI pigs (Figure 3A). Furthermore, we found that most of the AcCa-associated fatty-acyl chains were induced by adipocyte-specific UCP1 KI (Figures 3B–D). Notably, the concentration of SFA chains (C14:0, C15:0), MUFA chains (C10:1, C14:1, C16:1, C18:1) and PUFA chains (C14:2, C18:2, C20:3, C20:4, C22:6) displayed pronounced increases in UCP1 KI skeletal muscle (Figures 3B–D). In addition, the contents of SFA chains (C8:0, C16:0), MUFA chains (C22:1) and PUFA chains (C22:4) were significantly elevated in the KI group (Figures 3B–D). Moreover, we analyzed the total contents of SFA, MUFA, and PUFA chains associated with AcCa acyl chains. Adipocyte-specific UCP1 KI significantly augmented the MUFA percentage and greatly augmented the PUFA percentage; PUFA percentage was unaffected in the KI group (Figure 3E). Compared to skeletal muscle from the WT group, skeletal muscle from the UCP1 KI group displayed markedly elevated levels of AcCa species with higher (>13 carbons) acyl chain carbon numbers and fewer (<3 carbons) acyl chain double bonds (Figures 3F,G). These results suggest that adipocyte-specific UCP1 KI induces considerable alterations in the composition of AcCa species and acyl chains in skeletal muscle.

### Adipocyte-Specific Uncoupling Protein 1 Knock-in Altered the Transcriptional Profile of Skeletal Muscle

**FIGURE 4:**
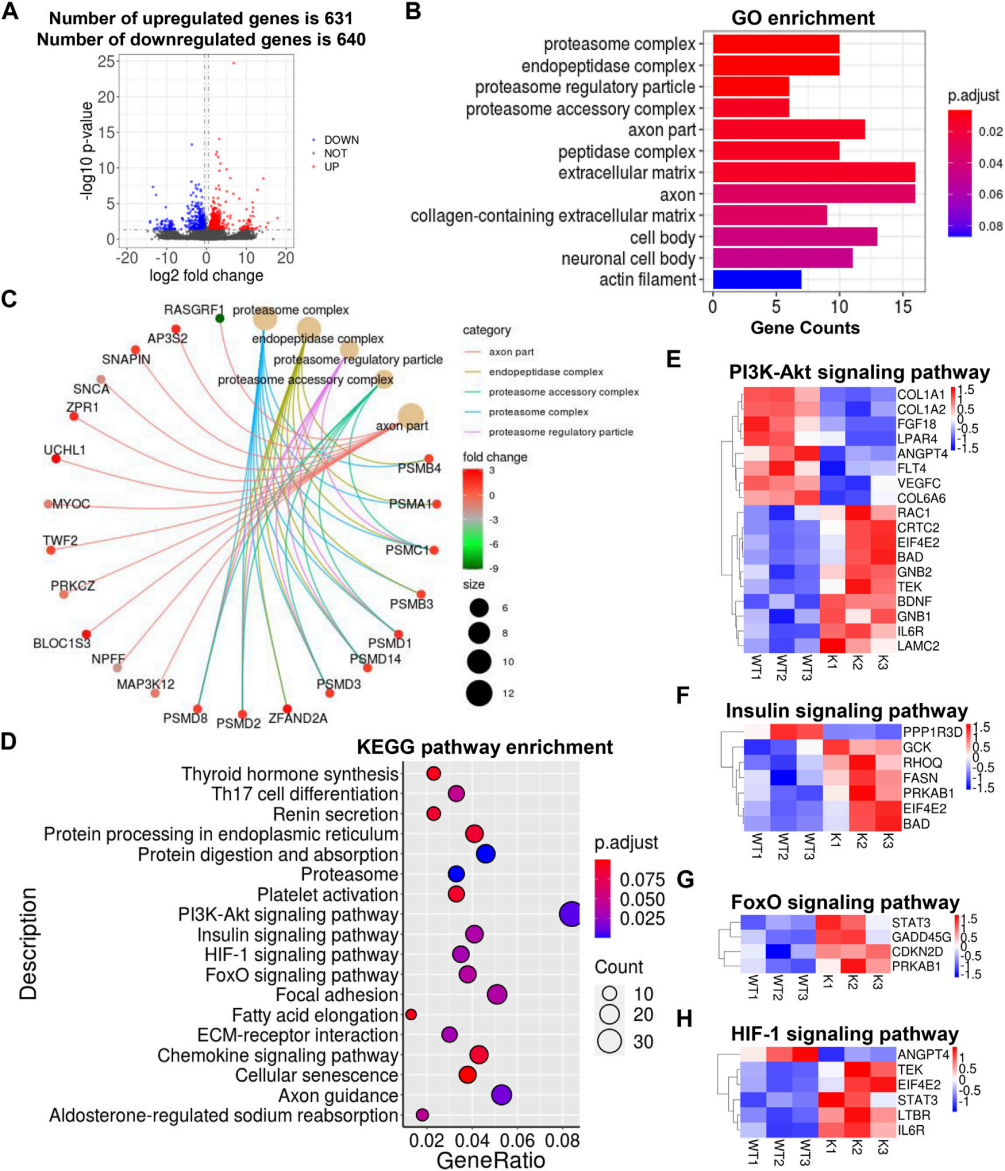
Alterations in skeletal muscle transcriptional profile by UCP1 KI. **(A)** Log2-fold changes in exons of RNA-seq gene bodies in skeletal muscle from UCP1 KI pigs versus controls and the corresponding significance values displayed as Log10 (*p*-value). The transverse and vertical dotted lines indicate the cutoff value for differential expression (*p* < 0.05 & Abs (Log2-fold changes) > 0.5). In total, 631 and 640 genes were identified that had induced (red) or repressed (blue) expression levels by cold exposure. **(B)** GO enrichment analysis and enriched terms were visualized by A bar plot. The bar color indicates significance, and the corresponding significance values are displayed as log10 (*p*-value). The bar length indicates significantly changed gene counts involved in certain categories. **(C)** The Cnetplot depicts the linkages of the five most enriched GO terms (proteasome complex, endopeptidase complex, proteasome regulatory particle, proteasome accessory complex and axon part) and genes involved in these terms as a network. The yellow dots indicate enriched GO terms and the size of each dot indicates gene counts involved in certain GO terms. These smaller dots indicate genes involved in these terms. The color of each smaller dot indicates log2(fold change) values genes in skeletal muscle from UCP1 KI pigs versus controls. **(D)** Functional enrichment analyses using KEGG pathways. The triangle size indicates gene counts. The dot color indicates significance and corresponding significance values displayed as log10 (*p*-value). **(E–H)** Heatmap of TPM expression values of metabolic pathways (including PI3K-Akt signaling pathway **(E)**, insulin signaling pathway **(F)**, FoxO signaling pathway **(G)** and HIF-1 signaling pathway **(H)**)-regulated genes from the RNA-seq dataset. Only genes with *p* < 0.05 are displayed.

To explore how the transcriptional profile in skeletal muscle changed upon adipocyte-specific UCP1 overexpression, we utilized RNA-seq to map the transcriptional changes and lipid metabolic pathways in skeletal muscle from UCP1 KI pigs. We identified a total of 1,271 differentially expressed genes (DEGs), of which 631 were upregulated and 640 were downregulated by UCP1 KI (Figure 4A). Our previous study reported the expression of foreign UCP1 was observed in iWAT of KI pigs but not in muscle or other tissues of these pigs (Zheng et al., 2017). Consistent with this observation, no expression of UCP1 mRNA was detected in KI or WT skeletal muscle by RNA-seq, and the other two mitochondrial uncoupling proteins, UCP2 and UCP3, were not significantly altered in skeletal muscle from UCP1 KI pigs, although UCP2 was increased (Supplementary Figure S4A). Adipocyte-specific UCP1 KI significantly increased the mRNA level of *CEBPB* without effects on other adipogenesis related genes (*FABP4*, *CEBPA* and *PPARG*) (Supplementary Figure S4B). We also found that the mRNA levels of intramuscular fat browning markers (*PPARA*, *BMP7*, *TNFRSF9*, *SHOX2*, *TMEM26*, *CITED1*) and mitochondrial function genes (*COX5A*, *COX6A2*, *COX6A1*, *TFAM*, *TFEB*) in skeletal muscle displayed no difference between the UCP1 KI and WT groups (Supplementary Figures S4C,D). Gene Ontology (GO) enrichment analysis of DEGs induced by UCP1 KI revealed pronounced changes in the proteasome complex, peptidase complex, extracellular matrix, axon part and neuronal cell body (Figure 4B). The Cnetplot depicted the linkages of five significant GO terms (proteasome complex, endopeptidase complex, proteasome regulatory particle, proteasome accessory complex and axon part) and genes involved in these terms as a network (Figure 4C). The significantly upregulated genes encoding proteasome components (*PSMC1*, *PSMD1*, *PSMD14*, *PSMD3*, *PSMD2*, *PSMD8*) belong to multiple categories, indicating that numerous cellular processes in skeletal muscle from UCP1 KI pigs might be regulated by the degradation of ubiquitinated target proteins through coordinated functions of genes associated with these four GO terms (proteasome complex, endopeptidase complex, proteasome regulatory particle, proteasome accessory complex) (Figure 4C). Axon part-related genes (*AP3S2*, *SNAPIN*, *BLOC1S3*, *Zrp1*), which coordinate with each other to regulate vesicles and signaling in neurons, were significantly increased in skeletal muscle from UCP1 KI pigs (Figure 4C), suggesting that motor neuron activity might be altered by adipocyte-specific UCP1 overexpression.

The above results showed that adipocyte-specific UCP1 KI in pig induces considerable alterations in the composition of lipid species in skeletal muscle, especially the contents of GLs and AcCas. Therefore, we analyzed several lipid biosynthesis pathways, including glycerolipid metabolism (*PLPP2*, *AKR1A1*, *AGK*), glycerophophoslipid metabolism (*PGS1*, *PLPP2*, *ETNPPL*) and sphingolipid metabolism (*CERS5*, *GBA*, *PLPP2*, *CERS3*), and produced heatmaps of differently expressed genes (Supplementary Figure S4E). Notably, the mRNA level of *PLPP2*, which functions in the glycerolipids-, glycerophophoslipid- and sphingolipid-metabolism pathways, was significantly induced by adipocyte-specific UCP1 overexpression (Supplementary Figure S4E). Heatmaps of differently expressed genes involved in fatty acid biosynthesis, fatty acid elongation and fatty acid degradation showed that the mRNA levels of fatty acid metabolism related genes (*MCAT*, *FASN*, *ELOVL3* and *ACADM*) were markedly increased by adipocyte-specific UCP1 overexpression, suggesting that fatty acid metabolism in skeletal muscle was more active in UCP1 KI pigs than in WT pigs (Supplementary Figure S4F).

We next performed Kyoto Encyclopedia of Genes and Genomes (KEGG) functional enrichment analyses (Kanehisa and Goto, 2000; Minoru et al., 2014) of the genes significantly induced by UCP1 KI and found significant enrichment of several major metabolic regulatory pathways (Figure 4D). Heatmaps showed significantly altered genes involved in the PI3K-Akt signaling pathway, insulin signaling pathway, HIF-1 signaling pathway and forkhead box O (FoxO) signaling pathway, which play critical roles in lipid metabolism (Figures 4E,F). Several genes (*ANGPT4*, *FLT4* and *VEGFC*) involved in PI3K-Akt signaling pathway and reportedly work together to receive epidermal growth factor signals and promote angiogenesis, were significantly decreased in the UCP1 KI pigs (Figure 4E). The *GCK*, which was upregulated in the KI pigs, is involved in insulin signaling pathway and reportedly plays roles in glucose metabolism and insulin secretion (Figure 4F). In addition, functional enrichment was found in the axon guidance, focal adhesion and chemokine signaling pathways, and significantly altered genes were evident in heatmaps (Supplementary Figures S4G–I). Some genes (*BAD*, *RAC1*, *STAT3*) that were significantly upregulated in the skeletal muscle tissues of UCP1 KI pigs are involved in multiple pathways and reportedly regulate cell growth and apoptosis in response to cytokines and growth factors (Figures 4E–H; Supplementary Figures S4G–I). These results indicated that ectopic UCP1 expression in iWAT induced a series of lipid metabolic regulatory pathways in skeletal muscle, suggesting a strong relationship between skeletal muscle and adipose tissue in lipid metabolism.

### Comparison of Genetic and Lipid Profiles in Adipose Tissue and Skeletal Muscle From Adipocyte-Specific Uncoupling Protein 1 Knock-in Pigs

**FIGURE 5:**
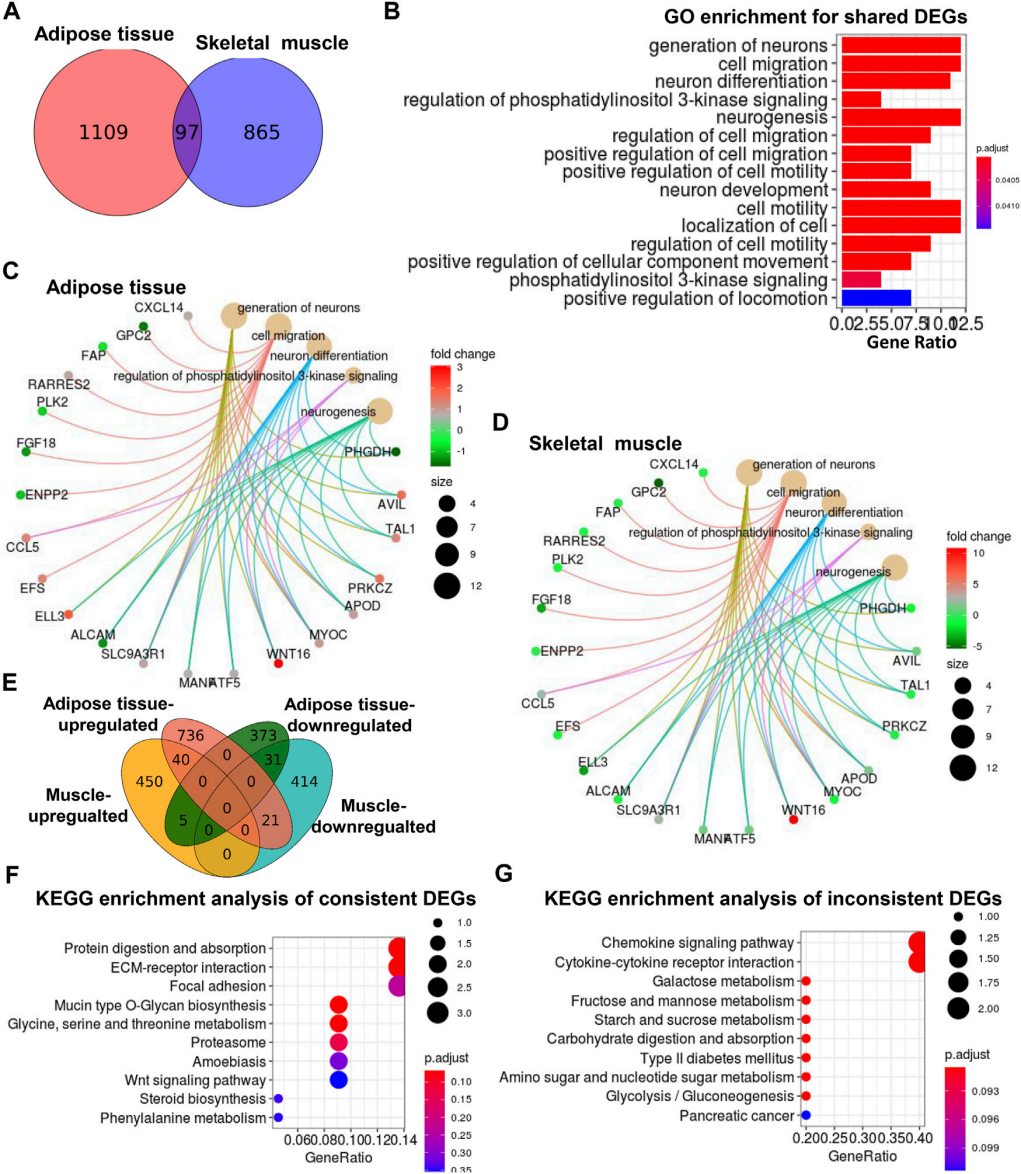
Comparison of significantly altered genes between adipose tissue and skeletal muscle from UCP1 KI pigs. **(A)** Venn diagram of the DEGs in adipose tissue and skeletal muscle between UCP1 KI pigs and WT pigs. **(B)** GO enrichment analysis of common DEGs was performed, and enriched terms were visualized by a bar plot. The bar color indicates significance and corresponding significance values displayed as log10 (*p*-value). The bar length indicates significantly changed gene counts involved in certain categories. **(C,D**) The Cnetplot depicts the linkages of the five most enriched GO terms and the genes involved in these terms as a network. The yellow dots indicate enriched GO terms and the size of each dot indicates gene counts involved in certain GO terms. These smaller dots indicate genes involved in these terms. The color of each smaller dot indicates log2(fold change) values genes in adipose **(C)** and skeletal muscle **(D)**, from UCP1 KI pigs versus controls. **(E)** Venn diagram of upregulated and downregulated DEGs in adipose and skeletal muscle between UCP1 KI pigs and WT pigs. **(F**,**G)** Functional enrichment analyses using KEGG pathways of shared consistent DEGs and inconsistent DEGs.

We compared the significantly altered genes and lipids between adipose tissue (Pan et al., 2019) and skeletal muscle from UCP1 KI pigs. As shown in the Venn diagram of adipose tissue and skeletal muscle, 97 common DEGs were screened in these two tissues (Figure 5A), and the log2(fold change) values of these DEGs in adipose and skeletal muscle were illustrated in heatmaps (Supplementary Figure S5A). GO enrichment analysis of the common DEGs revealed pronounced enrichment in terms associated with neuronal generation, cell migration, neuronal differentiation and phosphatidylinositol 3-kinase signaling regulation (Figure 5B). In addition, Cnetplots were constructed to depict the linkages of the top five significant GO terms and genes associated with these terms in adipose tissue (Figure 5C) and skeletal muscle (Figure 5D). Most genes involved in cell migration were regulated in the same direction in adipose tissue and skeletal muscle. In contrast, most of the genes involved in neuronal generation, neuronal differentiation and phosphatidylinositol 3-kinase signaling regulation were regulated in opposite directions in these two tissues (Figures 5C,D), suggesting that these shared functional pathways might be consistently or differently regulated by adipocyte-specific UCP1 ectopic expression between adipose tissue and skeletal muscle. Thus, we then analyzed the intersection of upregulated and downregulated DEGs of adipose and skeletal muscle tissue and found that 71 DEGs had consistent trends (including 40 upregulated DEGs and 31 downregulated DEGs) between adipose tissues and skeletal muscle, whereas 26 DEGs had opposite trends between these tissues (including five muscle-upregulated/adipose-downregulated DEGs and 21 muscle-downregulated/adipose-upregulated DEGs) (Figure 5E). KEGG functional enrichment analyses (Kanehisa and Goto, 2000; Minoru et al., 2014) based on the 71 shared DEGs with same trends revealed between the two types revealed enrichment in the pathways protein digestion and absorption, ECM-receptor interaction, and focal adhesion (Figure 5F). KEGG functional enrichment analyses (Kanehisa and Goto, 2000; Minoru et al., 2014) based on the 26 shared DEGs with opposite trends revealed enrichment in the pathways chemokine signaling and cytokine-cytokine receptor interaction (Figure 5G). These results supported the above hypothesis that neurons system function might contribute to the association between skeletal muscle and adipose tissue in the response to ectopic expression of UCP1 in iWAT. These results suggested that cytokine-mediated signaling might play unique roles in these two tissues in UCP1 KI pigs.

### Comparison of Lipid Biomarkers of Uncoupling Protein 1 Knock-in Pigs and Other Mammals

**FIGURE 6:**
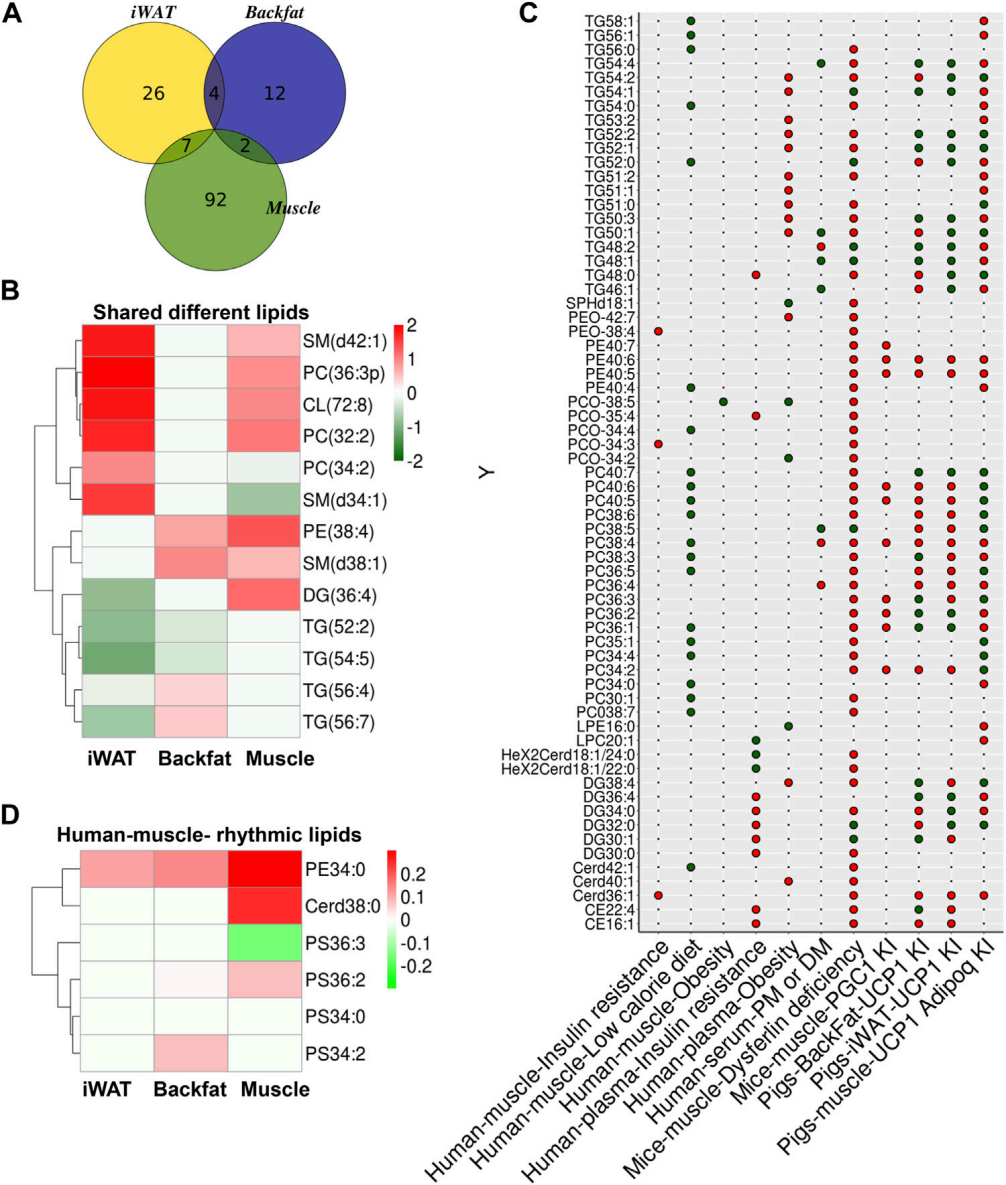
Comparison of lipid biomarkers from UCP1 KI pigs and other mammals. **(A)** Venn diagram of significantly altered lipids in iWAT, backfat and skeletal muscle between UCP1 KI pigs and WT pigs. **(B)** Heatmap of Log2(fold change) values of common significantly altered lipids of iWAT, backfat and skeletal muscle from UCP1 KI pigs. Red indicates high (2) Log2(fold change) values and blue indicates low (−2) Log2(foldchange) value. **(C)** The colors of the circles indicate trends of skeletal muscle and plasma lipid features from several published sources (insulin resistance in human muscle (Tonks et al., 2016), low calorie diet in human muscle (Nylén et al., 2019), obesity in human muscle (Tonks et al., 2016), insulin resistance in human plasma(Tonks et al., 2016), obesity in human plasma (Tonks et al., 2016), polymyositis and dermatomyositis (PM/DM) in human serum (Raouf et al., 2018), dysferlin deficiency in mice muscle (Haynes et al., 2019), and PGC1α KI in mice muscle (Senoo et al., 2015) and of iWAT, backfat and skeletal muscle from UCP1 KI pigs. Red indicates lipid features that showed increased trends in tissues from UCP1 KI pigs, and green indicates those with decreased trends. Black dots denote lipid features that were undetected in the lipidomics results of tissues from UCP1 KI pigs. **(D)** Heatmap of Log2(foldchange) values of lipids between human skeletal muscle and iWAT, backfat and skeletal muscle from UCP1 KI pigs. Red indicates high (0.3) Log2(fold change) value and blue indicates low (−0.3) Log2(fold change) values.

As lipids can function as signaling molecules, we compared the lipid profiles of adipose tissue and skeletal muscle from UCP1 KI pigs. The Venn diagram of skeletal muscle, iWAT and backfat showed seven significantly altered lipids that were shared between iWAT and skeletal muscle, two significantly altered lipids were shared between backfat and skeletal muscle, and four that were shared between backfat and iWAT (Figure 6A). A heatmap of the log2(fold change) values of these common significantly altered lipids in these three tissues was constructed (Supplementary Figure S5B). Some of these lipids (sphingomyelin (SM) (d42:1), PC (36:3p), CL (72:8), PC (32:2), SM (d38:1) and PE (38:4)) were increased in both skeletal muscle and adipose tissue (iWAT or backfat) f KI pigs relative to WT pigs (Figure 6B). In contrast, other lipids (SM (d34:1), PC (34:2) and DG (36:4)) had opposite trends between skeletal muscle and adipose tissue (iWAT or backfat) (Figure 6B). These shared lipids might function as signaling molecules between adipose tissue and skeletal muscle in UCP1 KI pigs.

Our previous studies reported that UCP1-KI pigs displayed improved thermoregulation upon cold exposure, greatly decreased fat deposition due to elevated lipolysis in iWAT (Zheng et al., 2017) and enhanced mitochondrial function in adipose tissue (Pan et al., 2019). In light of these findings, we wondered whether UCP1 KI pigs could be utilized to explore the regulatory mechanism of lipid metabolism in insulin resistance and obesity in humans. Thus, we compared the lipid profiles of these three tissues from UCP1 KI pigs with published data from several other sources, including lipidomic signatures of insulin resistance in human muscle (Tonks et al., 2016), low caloric diet in human muscle (Nylén et al., 2019), obesity in human muscle (Tonks et al., 2016), insulin resistance in human plasma (Tonks et al., 2016), obesity in human plasma (Tonks et al., 2016), polymyositis and dermatomyositis (PM/DM) in human serum (Raouf et al., 2018), dysferlin deficiency in mice muscle (Haynes et al., 2019), and PGC1α KI in mice muscle (Senoo et al., 2015; Figure 6C).

We found that some TGs (TG54:2, TG52:2, TG52:1, TG51:0, TG50:3, TG50:1) that which were increased by obesity in human plasma and by dysferlin-deficiency in mice skeletal muscle were decreased in iWAT, backfat or skeletal muscle from UCP1 KI pigs, suggesting that the increase of TGs by obesity or dysferlinopathy might be reversed by UCP1 ectopic expression (Figure 6C). Some TGs (TG58:1, TG56:1, TG54:0, TG52:0) showed inverse trends between human muscle associated with a low-calorie diet and skeletal muscle from UCP1 KI pigs, suggesting that the mechanism of lipid metabolism alteration might differ between these two tissues (Figure 6C). The lipid biomarkers of insulin resistance in human muscle or plasma (TG48:0, PEO-38:4, PE40:7, PCO-35:4, PCO34:3, DG34:0, DG30:0, Cerd36:1, CE22:4, CE16:1) were increased in the skeletal muscle of dysferlin-deficient mice, and some of these lipids (TG48:0, DG34:0, CE22:4) showed inverse patterns in adipose or skeletal muscle from UCP1 KI pigs, suggesting that insulin resistance might be strongly correlated with dysferlinopathy and that UCP1 ectopic expression might have a potential effect on protecting against the lipid dysfunction induced by insulin resistance or dysferlinopathy (Figure 6C). Some phospholipids (PE40:6, PE40:5, PC38:4, PC36:2, PC36:1) altered by overexpression of PGC-1α in skeletal muscle showed the same trends in skeletal muscle from UCP1 KI pigs, while other (PC40:6, PC40:5, PC36:3, PC34:2) showed opposite trends between PGC-1α-overexpressed skeletal muscle and skeletal muscle from UCP1 KI pigs (Figure 6C). We also found differences between human skeletal muscle and skeletal muscle from UCP1 KI pigs (Figure 6D). Some lipids (PE34:0, Cerd38:0, PS36:2, PS34:2) were increased in adipose or skeletal muscle from UCP1 KI pigs, while PS36:3 was decreased in skeletal muscle from UCP1 KI pigs (Figure 6D). These results show that adipose tissue and skeletal muscle from UCP1 KI pigs shared some lipid features. Furthermore, they indicate that UCP1 KI pigs might be serve as a potential model for lipid metabolism-based explorations of the regulatory mechanisms of some metabolic disorders and myopathy in humans, especially insulin resistance and dysferlinopathy.

## Discussion

In this study, we utilized LC-MS/MS -based lipidomics and RNA-seq to extend our understanding of alterations of lipid metabolism and the associated pathways in skeletal muscle from UCP1 KI pigs. Our results showed that UCP1 KI induced significant changes in the contents of specific lipids, the overall composition of lipid classes, and the length of acyl chains associated with GLs and sphingolipids in skeletal muscle. UCP1 KI induced many DEGs enriched in several major metabolic regulatory pathways. Our results revealed that UCP1 KI induced lipid dynamics and gene programs in skeletal muscle and provided lipidomic and transcriptional signatures of skeletal muscle from UCP1 KI pigs.

Using untargeted lipidomics, we found that several lipid classes in skeletal muscle, including PGs, LPGs, LPCs, CLs, AcCas, and SQDGs, are remodeled selectively by UCP1 KI. In our previous study, we found that compared to that from WT pigs, iWAT from UCP1 KI pigs displayed significantly decreased levels of TGs, increased levels of total sphingolipids and markedly increased levels of two CL species (Pan et al., 2019). The distinct lipidomic profiles of adipose and skeletal muscle indicated that lipid metabolism in these two tissues was regulated in a tissue-specific manner by adipose-specific UCP1 KI. This finding is consistent with the different composition and functions of adipose tissue and skeletal muscle. Nevertheless, the total content of mitochondrial-specific lipid species, CLs, displayed similar increased trend in adipose tissue (*p* = 0.064) and skeletal muscle (*p* = 0.05). In particular, the contents of CL72:8 (18:2) and CL74:9 (18:2) in adipose tissue and those of CL (18:2/18:2/18:2/18:2) in skeletal muscle were markedly increased by adipocyte-specific UCP1 KI. CLs contribute to mitochondrial membrane stability and dynamics and are required for numerous mitochondrial activities, especially those related to oxidative phosphorylation and coupled respiration (Paradies et al., 2010; Unsay et al., 2013). In brown and beige fat, CLs bind directly to UCP1, increasing its stability in the mitochondrial membrane (Lee et al., 2015), and they play essential roles in systemic energy homeostasis (Sustarsic et al., 2018). Thus, the activation of CL biosynthesis and mitochondrial function in both adipose and skeletal muscle might account for the improved thermogenesis upon cold exposure in UCP1 KI pigs (Pan et al., 2019; Zheng et al., 2017). However, pigs do not have classical BAT and the reconstruction of UCP1 is done in adipocytes that are more likely similar to white adipocytes with minimal number of mitochondria. Ectopic expression of UCP1 protein by the KI procedure may not have physiological milieu to perform the conventional function of proton-gradient-dissipation inside mitochondrial inner membrane in this unnatural condition. Hence, the specific localization of the overexpressed UCP1 inside white adipocytes of pigs needs to be further explored. Furthermore, we found that the total content of PGs that are used to synthesize CL was significantly increased in skeletal muscle from UCP1 KI pigs. Cold exposure-induced lipid signature of PGs in serum/plasma samples might be associated with the activated phosphatidylglycerol/CL metabolism in both BAT and WAT (Lynes et al., 2018). Although, the circulating lipid profiles of UCP1 KI pigs remain unexplored, our previous study revealed significantly decreased TGs and increased FFAs in the serum of UCP1 KI pigs relative to the serum contents in WT pigs (Zheng et al., 2017). Indeed, lipids released by adipose, such as FFAs and CLs, can act as messenger molecules that affect the metabolism and function of skeletal muscle (Ren et al., 2014; Tomas et al., 2004; Zhou et al., 2007). Therefore, we hypothesized that the coordinately increased CL lipid species in the adipose tissue and skeletal muscle of UCP1 KI pigs might interact with each other to remodel the circulating lipid profile. Cytokines might also transmit signals between adipose and skeletal muscle, as suggested by the enrichment in the KEGG pathways chemokine signaling pathway and cytokine-cytokine receptor interaction of shared DEGs observed between adipose tissue and skeletal muscle from UCP1 KI pigs.

Previous research showed that compared with WT pigs, UCP1 KI pigs displayed a significant increase of circulating FFAs (Zheng et al., 2017). Such an increased, might regulate insulin resistance in human skeletal muscle through the inhibition of glucose transport activity, a consequence of decreased IRS-1-associated PI3-kinase activity (Dresner et al., 1999). We found that the lipid class with the most species upregulated by adipose-specific UCP1 ectopic expression in skeletal muscle was AcCas. Mitochondrial overload and incomplete muscle fatty acid β-oxidation induced by metabolic situations of high FFA availability results in AcCas accumulation (Koves et al., 2008; Gavin et al., 2018), which might interfere with oxidative stress and insulin resistance (Schooneman et al., 2013; Aguer et al., 2015). We also found that the skeletal muscle content of diglyceride (DG) (36:4) and ceramide (Cer) (d18:2/20:0) were significantly increased in UCP1 KI pigs, while the total contents of DGs and Cers in skeletal muscle did not differ between UCP1 KI and WT pigs. The relationships of DGs and Cers with insulin resistance are debated (Dube et al., 2011; Devries et al., 2013; Sogaard et al., 2016), the roles of individual lipids require elucidation. The RNA-seq results revealed enrichment in the KEGG pathway insulin signaling pathway and several oxidative stress related pathways, including the PI3K-Akt signaling pathway, HIF-1 signaling pathway and FoxO signaling pathway. The network of these regulatory pathways reportedly plays key roles in cell progress and glucose metabolism and critical energy functions in skeletal muscle (Cazarolli et al., 2013; Schiaffino et al., 2013; Görgens et al., 2017; Timmer et al., 2018). However, investigations of insulin resistance, glucose transport rate, glucose phosphorylation activity, and other processes are required to confirm the insulin-resistance and glucose-metabolism alterations in skeletal muscle and explore the mechanism by which UCP1 KI induce lipid alterations and metabolic dysfunction.

Numerous studies have demonstrated the recruiting and functions of human brown fat by applying cold exposure treatment to the adult body. Cold acclimation has been advised as an acceptable and low-cost method to increase energy expenditure and counteract the current obesity epidemic (van der Lans et al., 2013). BAT mitochondrial UCP1 and skeletal muscle thermogenesis provides the primary mechanism for temperature homeostasis to defend body temperature at cold temperatures. Based on our current results, we found that UCP1 KI induces changes in skeletal muscle metabolism. Hence, cold acclimation of UCP1-KI pigs may bring greater alterations in the skeletal muscle, the regulatory network and metabolic changes need to be further explored. To explore the potential of adipose-specific UCP1 KI pigs as models of human metabolic diseases, we compared the lipid profiles of three tissues from UCP1 KI pigs with several other published studies of metabolic disorders and muscle disease (Senoo et al., 2015; Tonks et al., 2016; Raouf et al., 2018; Haynes et al., 2019; Nylén et al., 2019). We found that the lipid biomarkers in UCP1 KI pigs had the highest correlations with lipid biomarkers in human insulin resistance and mice dysferlinopathy. Peroxisome proliferator-activated receptor gamma coactivator 1α (PGC-1α) is central to mitochondrial biogenesis and functioning (Yeo et al., 2019), and significantly increased contents of GLs were observed in both PGC-1α-overexpressed skeletal muscle and skeletal muscle from adipocyte-specific UCP1-KI pigs. Furthermore, the correlations of lipid biomarkers in skeletal muscle between skeletal muscle-specific PGC1a KI mice and adipocyte-specific UCP1 KI pigs suggested that the lipid metabolism alterations in skeletal muscle from UCP1 KI pigs might be regulated by factors other than mitochondrial influence, such as adipokines. Future studies are needed to determine the regulation mechanism in this model.

In addition to the interplay skeletal muscle and adipose tissue, the adipocyte communicates with other organs to adjust energy balance and maintain metabolic homeostasis through fatty acids and adipokines (Stern et al., 2016). Indeed, adipocyte-derived secretory factors have been reported to play a role in maintaining glucose, lipid, and energy homeostasis. In our study, UCP1 KI induces a variety of positive and negative changes in muscle metabolism. However, whether these changes are mediated by UCP1 KI adipocytes-derived adipokines and growth factors remains to be further explored. Furthermore, KI-induced alteration of other tissues may also be there too due to the crosstalk with other organs, like brain, liver and pancreas. It will be a very meaningful and promising work to explore the changes in adipokines and fatty acids caused by adipocyte-specific UCP1 KI.

## Conclusion

In conclusion, our lipidomics data revealed alterations in the lipid profile in skeletal muscle induced by exogenous UCP1 expression in adipocytes, which reflect the changes in fatty acid contents and composition of meat from adipocyte-specific UCP1 KI pigs. The use of adipocyte-specific UCP1 KI pig generated using CRISPR/Cas9-mediated site-specific integration provides a transgenic strategy to improve fat traits in pigs. Furthermore, UCP1 KI pigs represent a model for understanding metabolic disorders and for discovering, validating and optimizing novel therapeutics.

## Data Availability

The data presented in the study are deposited in the NCBI Gene Expression Omnibus (GEO) repository, accession number GSE189334.
