# Supplementary material for: UCP1 Knockin Induces Lipid Dynamics and Transcriptional Programs in the Skeletal Muscles of Pigs
Source: Front Cell Dev Biol. 2022 Jan 12;9:808095. doi: 10.3389/fcell.2021.808095 (PMC8790096; doi:10.3389/fcell.2021.808095)

**Supplementary Figure 1.** **Multivariate data analysis of LC-MS lipidomics data from skeletal muscle samples from WT and UCP1 KI pigs.** (**A**) Quantified lipid classes and their abbreviations used throughout the paper. (**B**) Unsupervised PCA score plot. (**C**, **D**) Supervised PLS-DA and OPLS-DA models. Blue and green symbols represent skeletal muscle samples from WT and UCP1 KI pigs, respectively.

**
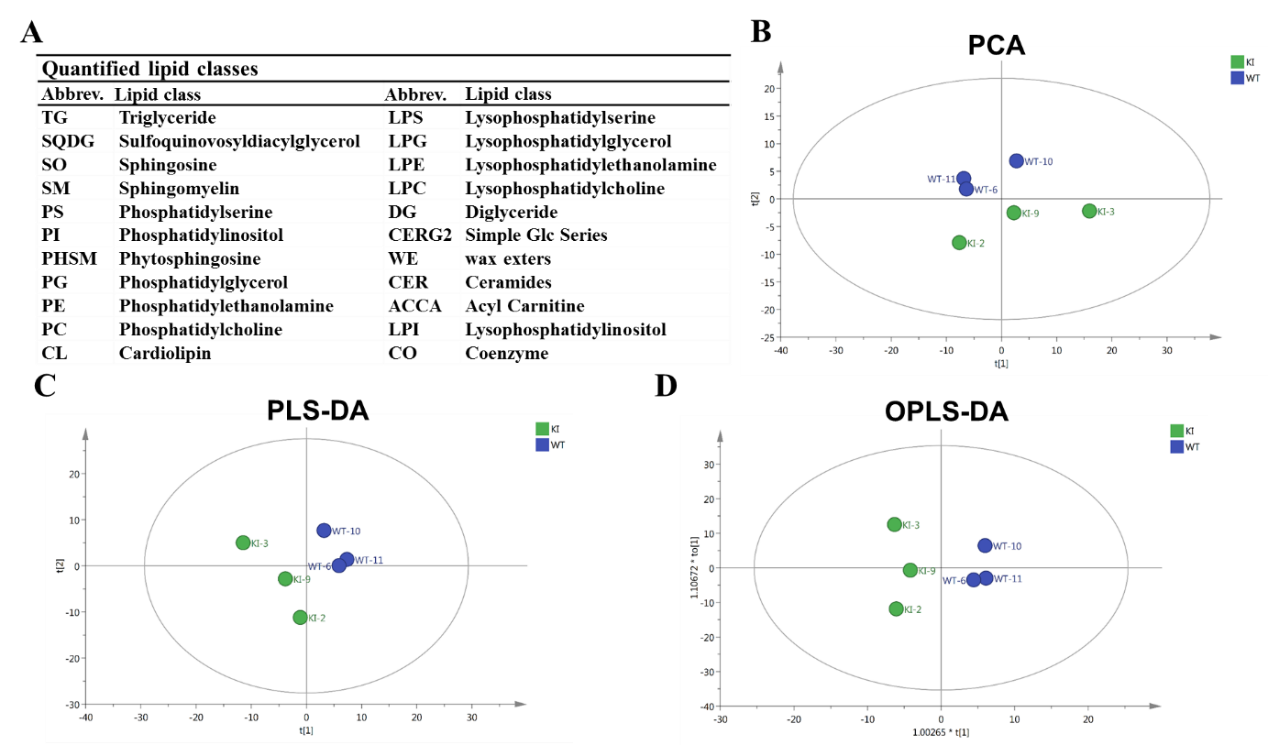
**

**Supplementary Figure 2. Correlations of lipid features.** The correlation matrix of these 72 significantly changed lipid species, with P-values <0.05 and VIP>1, was calculated by the function rcorr() of the R package Hmisc and the visualized correlogram was plotted using the function corrplot() of the R package corrplot. The dotted color indicates the correlation coefficient; blue indicates a positive correlation (1) and red indicates a negative correlation (-1).

**
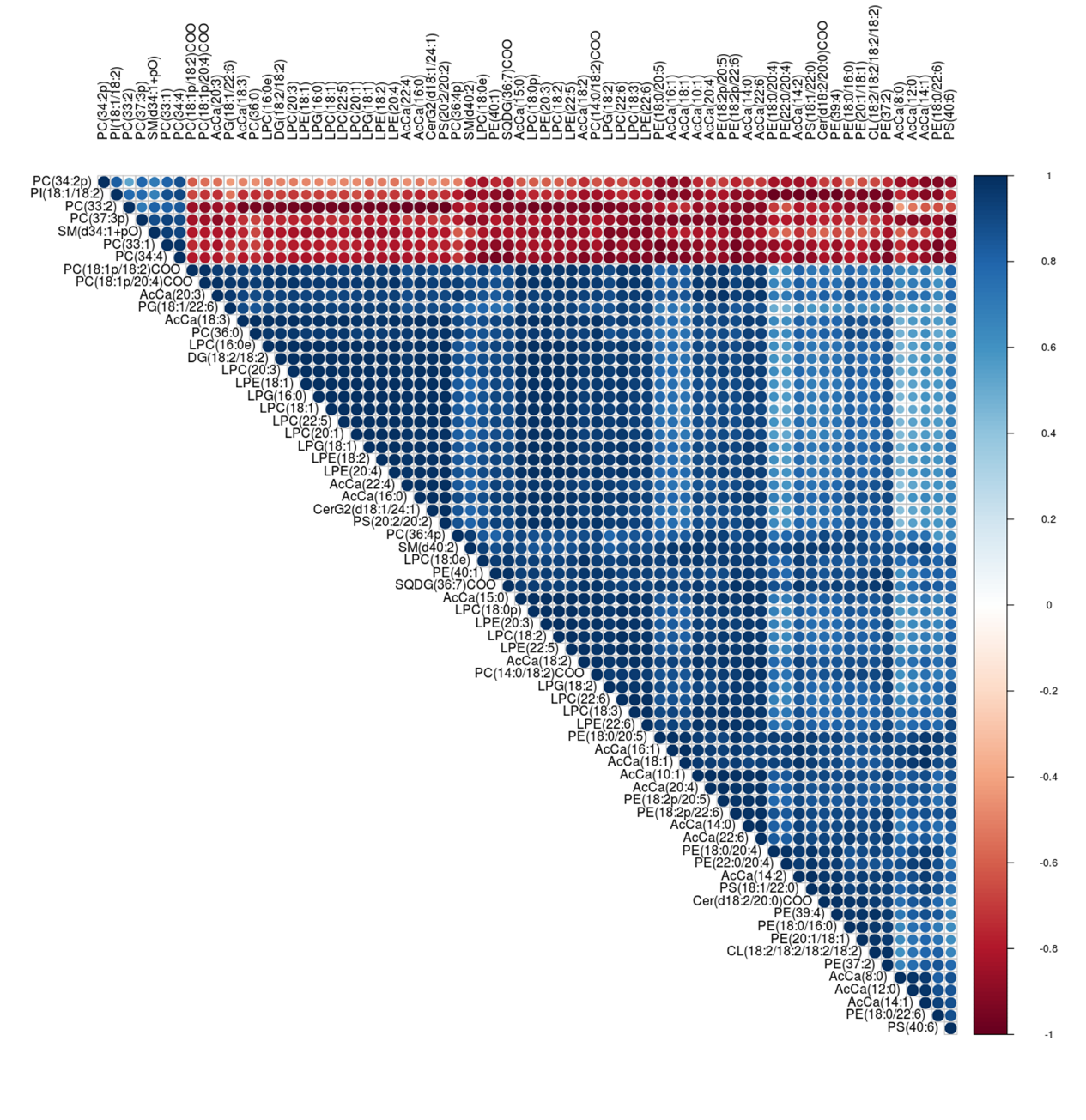
**

**Supplementary Figure 3. UCP1 KI did not change the composition of fatty acyl chains associated with TGs in skeletal muscle.** (**A-D**) The total lipid ion intensity of individual fatty acyl chains associated with TGs sorted by degree of intensity. (**E**) The total percentages of SFA chains, MUFA chains and PUFA chains associated with TGs. SAF, saturated fatty acyls; MUFA, monounsaturated fatty acyls; PUFA, polyunsaturated fatty acyls containing two or three to six double bonds; ODD, odd-numbered fatty acyls. Data are presented as means ± SEM (n = 3). * P < 0.05, *** P < 0.001. (**F, G**) The TG pattern in UCP1 KI cases versus that in controls. Each dot or triangle represents a distinct TG, organized along the x-axis based on the total acyl chain carbon number (**F**) or double bond content (**G**). The size of each dot or triangle is proportional to the significance values, which are displayed as -log10 (P-value).

**
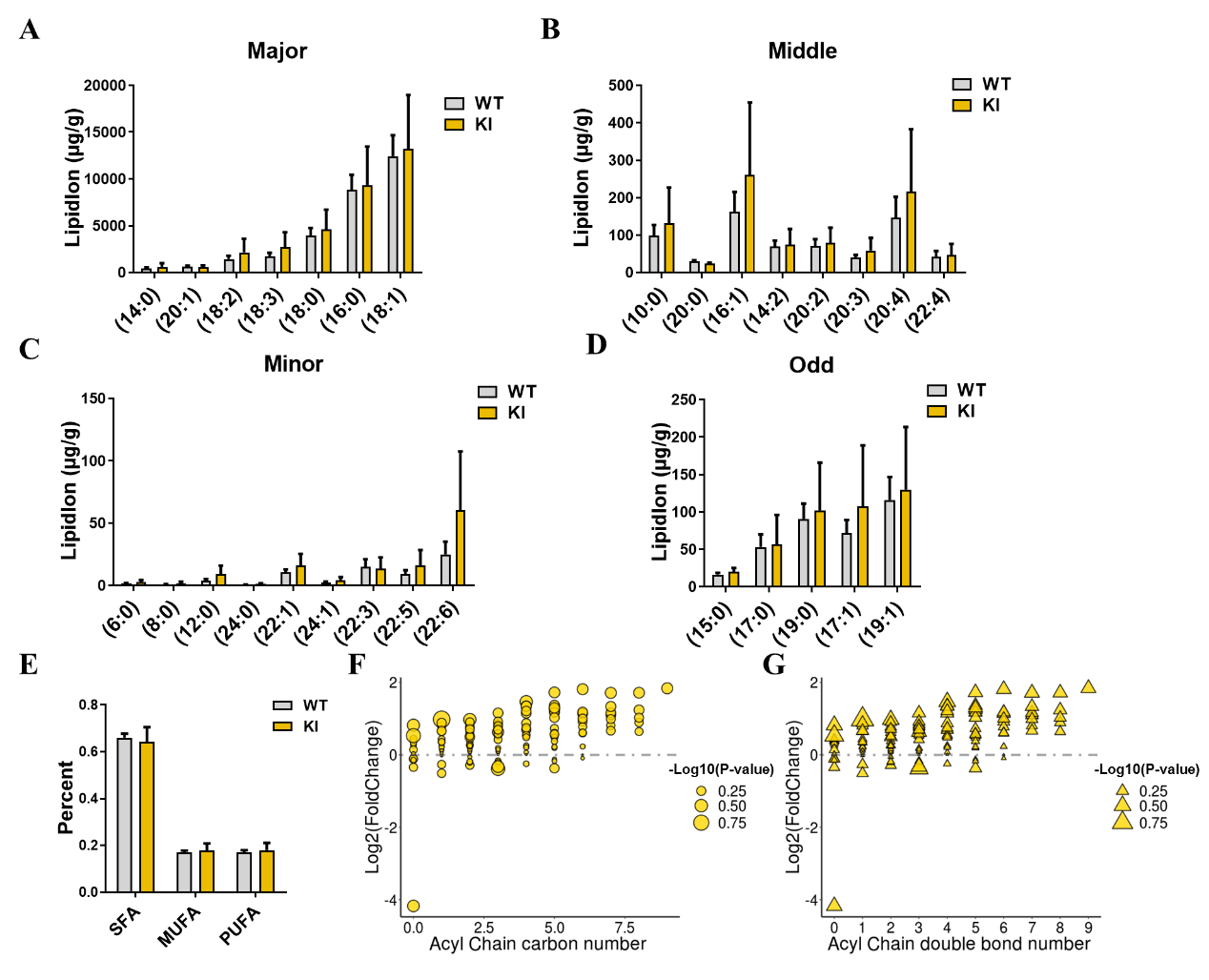
**

**Supplementary Figure 4. Expression of mitochondrial function genes, FoxO family genes and fatty acid metabolism-related genes in skeletal muscle from UCP1 KI pigs.** (**A**) TPM expression values of UCP2 and UCP3 in skeletal muscle from the two cases were shown to estimate relative gene expression abundance. (**B, C**) TPM expression values of adipogenesis (**B**) and thermogenesis (**c**) related genes in skeletal muscle from the two cases are shown. (**D**) TPM expression values of mitochondrial function-related genes in skeletal muscle from the two cases are shown. Data are presented as means ± SEM (n = 3). * P < 0.05, *** P < 0.001. (**E**) Heatmap of the transcripts per million (TPM) expression values of selected glycerolipid, GL and sphingolipid metabolism-regulated genes from the RNA-seq dataset. Only genes with P < 0.05 are displayed. (**F**) Heatmap of TPM expression values of selected fatty acid biosynthesis-, fatty acid elongation- and fatty acid degradation-related genes from the RNA-seq dataset. Only genes with P < 0.05 are displayed. (**G-I**) Heatmap of TPM expression values of other enriched pathways (including axon guidance, focal adhesion, and chemokine signaling pathway)-regulated genes from the RNA-seq dataset. Only genes with P < 0.05 are displayed.


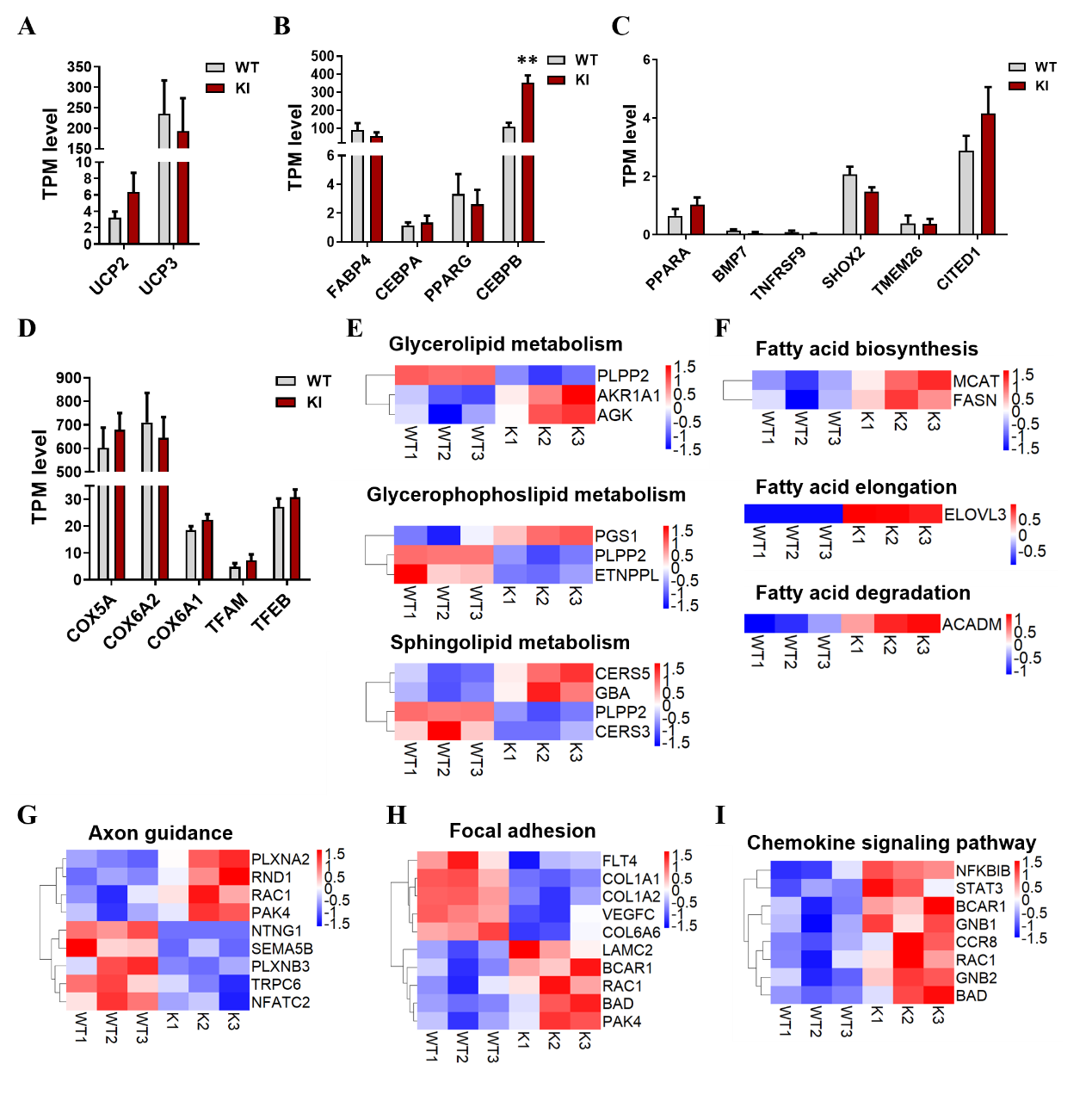


**Supplementary Figure 5. Comparison of significantly altered genes and lipids between adipose tissue and skeletal muscle from UCP1 KI pigs.** (**A**) Heatmap of log2(fold change) values of common DEGs of adipose tissue and skeletal muscle from UCP1 KI pigs. Red indicates a high (6) log2(fold change) value, and blue indicates a low (-6) log2(fold change) value. (**B**) Heatmap of log2(fold change) values of all significantly altered lipids of adipose tissue and skeletal muscle from UCP1 KI pigs. Red indicates a high (4) log2(fold change) value, and blue indicates a low (-4) log2(fold change) value.


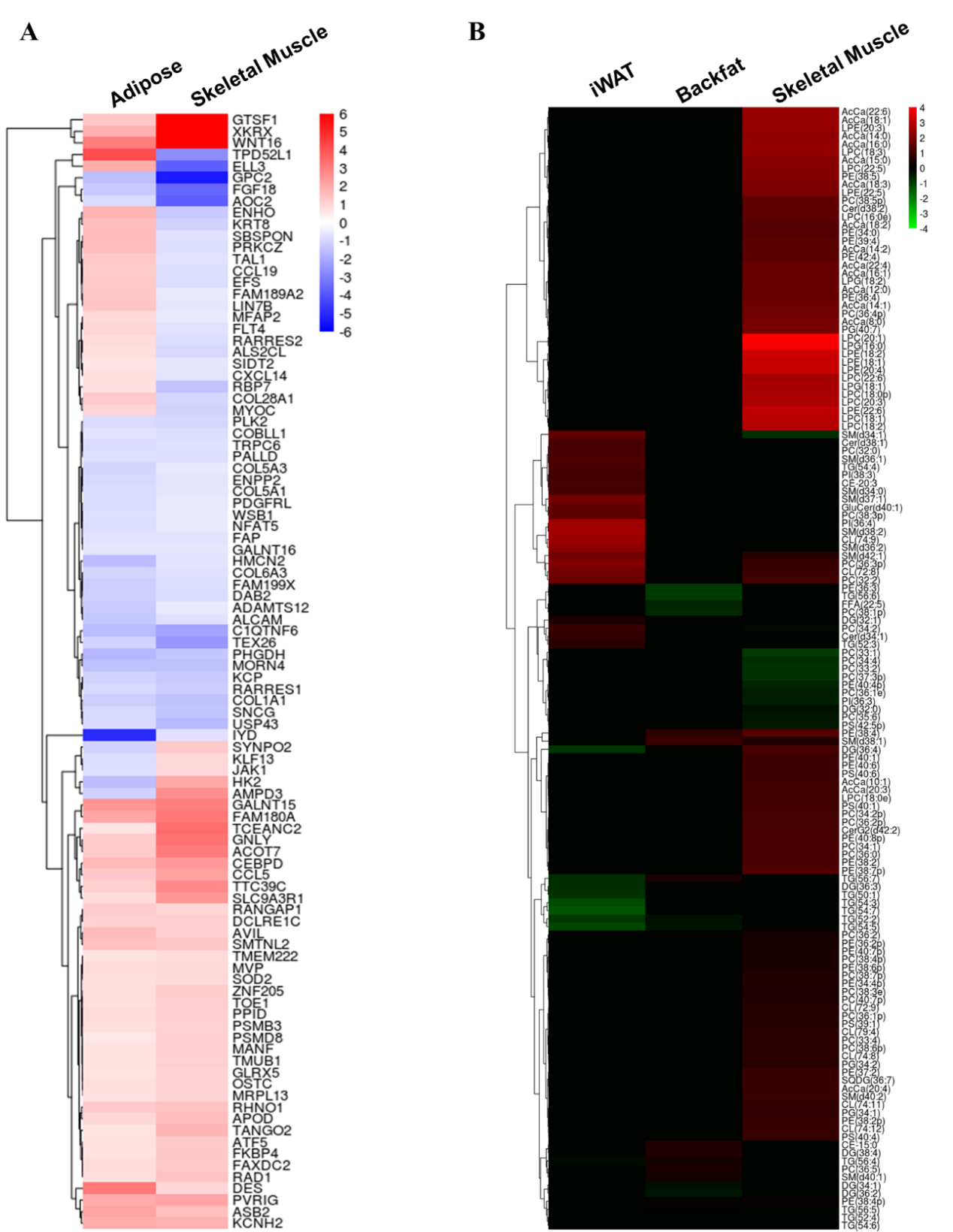

Supplement: Supplementary file 1 [file DataSheet1.docx]
